# Coexistence of an aberrant right subclavian artery and anomalous origins of bilateral vertebral arteries: A case report

**DOI:** 10.1097/MD.0000000000025043

**Published:** 2021-03-05

**Authors:** Yihong Wu, Huayi Zhang, Chenye Tang

**Affiliations:** a Department of Ultrasonography, Yuxin Central Hospital; b Department of Vascular Surgery; c Department of Urology, The Second Affiliated Hospital of Jiaxing University, Jiaxing, China.

**Keywords:** aberrant right subclavian artery, aortic arch, case report, variation, vertebral artery

## Abstract

**Rationale:**

Anatomical variations in aortic arch (AA) branching are not unusual. Generally, these variations are asymptomatic and are diagnosed incidentally. Here, we report a rare case of a middle-aged female patient with an aberrant right subclavian artery (ARSA) associated with anomalous origins of the bilateral vertebral arteries (VAs).

**Patient concerns:**

The patient treated for urolithiasis complained of repeated dizziness for several years.

**Diagnoses:**

Echocardiography and computed tomography angiography (CTA) confirmed arterial variations. Moreover, mild stenosis was found in the left common carotid artery (LCCA), which was considered to be the cause of dizziness.

**Interventions:**

Congenital anomalous arteries were not necessary to intervene urgently, but aspirin and atorvastatin were administered to prevent potential thrombosis attributed to vascular stenosis after completing the operation for urolithiasis.

**Outcomes:**

Whether the symptoms will be alleviated or not should be continuously followed up, and the patient may accept interventional therapy in the future if necessary.

**Lessons:**

Here, we report the rare variation of AA branches and highlight the importance of preoperative vascular assessment in surgical or interventional procedures for the affected body regions.

## 1 Introduction

The standard anatomy of the aortic arch (AA) includes 3 branches arising from its superior border from right to left and includes the innominate artery (IA), which then branches into the right subclavian artery (RSA) and the right common carotid artery (RCCA), the left common carotid artery (LCCA), and the left subclavian artery (LSA). As an essential source of blood supply to the posterior circulation, the vertebral artery (VA) usually arises from the posterosuperior aspect of the first part of the ipsilateral subclavian artery (SA). It then travels obliquely to enter the transverse foramen of the C6 vertebra. According to several large-sample studies, 65.9% to 79.2% of the population had a typical AA branching pattern and VA origin, whereas 20.8% to 34.1% had variations.^[1–3]^ Aberrant right subclavian artery (ARSA) is one of the most abundant AA branching anomalies, with an incidence rate of 0.5% to 1.3%.^[2,3]^ Anomalous origin of VA is more frequent on the left (6.0%) than on the right side (3.8%),^[4]^ and quite rarer on the bilateral side (approximately 3.0% of all the patients with anomalous origin of VA).^[5]^ Herein, we present the case of a patient with an ARSA associated with anomalous origins of bilateral VAs.

## 2 Case presentation

**Figure 1:**
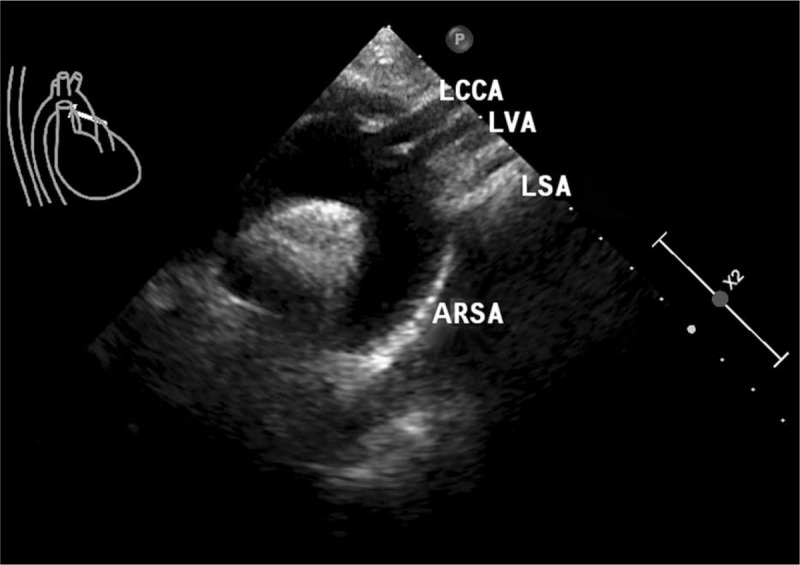
Echocardiography showing the variations of AA branches.

**Figure 2:**
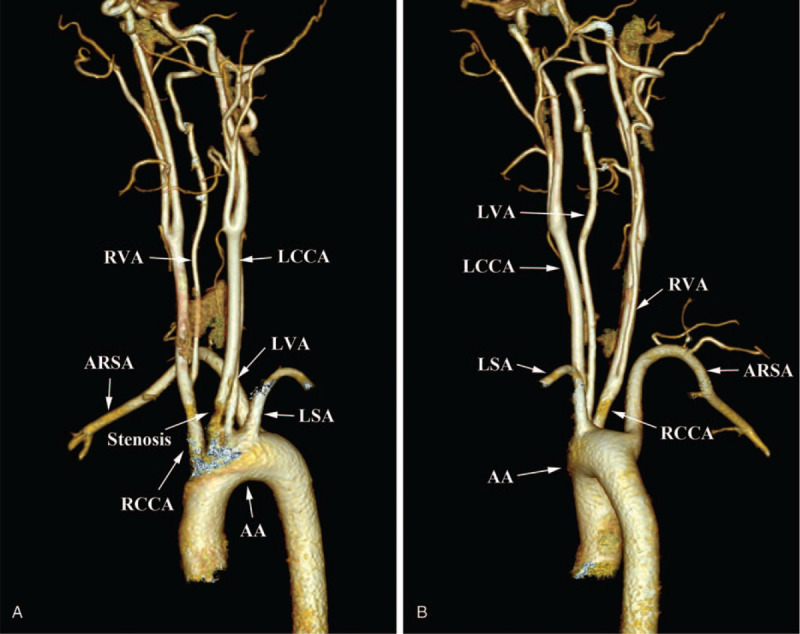
CTA showing the variations of AA branches and the stenosis in LCCA. (A). The 5 branches of the AA (anterior view) and the stenosis in LCCA. (B). The 5 branches of the AA (left lateral view) and the origin of RVA from the RCCA.

A 43-year-old female patient was treated for urolithiasis in the Department of Urology, who complained of repeated dizziness for several years. Preoperative echocardiography showed an ARSA derived from the initial descending aorta right rear to the LSA and an aberrant left vertebral artery (LVA) that directly originated from the AA instead of the LSA (Fig. 1). Then, computed tomography angiography (CTA) was performed, not only revealing the aforementioned variations of the arteries but also confirming an aberrant right vertebral artery (RVA) that originated from the RCCA rather than the RSA (Fig. 2A, B). The diameters of the initial segment of ARSA, LVA, and RVA were about 8.35, 4.14, and 3.22 millimeters, respectively, and no stenosis was seen in any course of these 3 arteries. The ARSA run behind the trachea and esophagus to the right side of the body; the LVA and RVA ascended upward and then entered the foramen transversarium of the C4 and C5 vertebrae, respectively. Moreover, mild stenosis was found in the proximal portion of the LCCA, adjacent to its aortic origin (Fig. 2A). No other cardiac or vascular anomalies were found on the above-mentioned imageological examinations. To prevent potential thrombosis attributed to vascular stenosis, aspirin and atorvastatin were used after completing the operation for urolithiasis, and the patient was followed up continuously. This study was approved by the Ethics Committee of the Second Affiliated Hospital of Jiaxing University, and written informed consent was obtained from the patient.

## 3 Discussion and conclusions

Variations in AA branching are usually asymptomatic and detected accidentally by autopsy^[6,7]^ or imagological examinations performed for other causes.^[8,9]^ In the study by Karacan et al,^[3]^ the AA branching patterns were categorized into 7 types, not including the form of our patient. Only an ARSA was defined as type 5 variation, while coexistence of ARSA and bicarotid trunk was classified as type 6 variation, and their incidences were 0.6% and 0.7%, respectively; meanwhile, the frequency of these 2 types was higher among females than among males.^[3]^ The ARSA results from the embryological degeneration of the right dorsal aorta above the seventh intersegmental artery (generally below the seventh intersegmental artery).^[10]^ The majority of ARSAs (80%) run behind the esophagus and can sometimes cause compression of the esophagus, making it difficult to swallow.^[11]^ Less frequently, the ARSA may travel between the esophagus and trachea (15%) or before the trachea (5%), potentially resulting in cough or airway obstruction.^[11]^ When a bulb-like swelling is present in the proximal portion of the SA near its origin, it is called a Kommerell diverticulum. Although uncommon, it may occur in approximately 60% of patients with ARSA.^[12]^ Once it occurs, the symptoms mentioned above may be more prominent. Additionally, catastrophic consequences may occur, such as distal embolism and diverticulum rupture. Therefore, such cases should be actively treated by surgery, if possible. Fortunately, Kommerell diverticulum was not present in our patient.

The LVA directly originating from the AA between the LCCA and LSA seen in our patient is the most common variation of VA origin^[5,8]^ and was defined as type 3 variation in the series categorized by Karacan et al.^[3]^ If combined with IA and LCCA arising from the AA in a common trunk, the AA branching pattern was categorized as type 4 variation, and the incidence rates of type 3 and 4 variations were 4.1% and 1.2%, respectively.^[3]^ Variations in RVA origin are relatively rare, and the most common is an RVA originating from the extreme proximal RSA; fewer cases of RVA arising from the RCCA are seen in patients with ARSA, with a reported incidence rate of 0.1%.^[13]^ This rare variation in arteries was also observed in this case. Embryologically, the first or second intersegmental artery failing to regress generates an abnormal origin of the VA from the internal or external carotid, while the permanent artery occurring from the third to sixth intersegmental artery results in an aberrant VA arising from the AA or common carotid artery.^[10]^ Variations in VA origin do not result in clinical symptoms; however, a direct aortic origin of the VA may cause hemodynamic changes that may lead to related complications. Compared with the classic subclavian origin of either the LVA or RVA, an aortic origin of the LVA showed an increased risk of aortic dissection,^[14]^ and it may be an independent risk factor for arterial dissection.^[15]^ Some researchers have postulated that a longer extracranial course may make the vessel wall more vulnerable to shear force, which may lead to intimal tear and dissection.^[15]^ Therefore, if headache and neurological symptoms occur suddenly in a patient with an anomalous VA originating from the AA, a thorough examination should be performed. It is not clear whether VA arising from the common carotid artery would increase the rate of complications when compared with the normal subclavian origin of VA. Our patient had dizziness, which was thought to have no connection to the anomalous origins of the VAs. It is possible that stenosis of the LCCA was accountable. Conservative medication and long-term follow-up rather than operations were recommended because the arterial stenosis was partial and mild.

In conclusion, the coexistence of an ARSA and anomalous origins of bilateral VAs is an infrequent condition. Misidentification of variant branches may lead to life-threatening complications during surgical or endovascular procedures. Therefore, careful examination and advance knowledge of the anatomic variants are essential for scheduling surgical or interventional procedures involving the thorax, neck, and head.

## Author contributions

**Case discussion and interpretation:** Huayi Zhang.

**Conceptualization:** Yihong Wu, Chenye Tang.

**Validation:** Yihong Wu, Huayi Zhang, Chenye Tang.

**Writing – original draft:** Yihong Wu.

**Writing – review & editing:** Huayi Zhang, Chenye Tang.
